# Consolidation of Spray-Dried Amorphous Calcium Phosphate by Ultrafast Compression: Chemical and Structural Overview

**DOI:** 10.3390/nano14020152

**Published:** 2024-01-10

**Authors:** Sylvain Le Grill, Christophe Drouet, Olivier Marsan, Yannick Coppel, Vincent Mazel, Marie-Claire Barthelemy, Fabien Brouillet

**Affiliations:** 1 CIRIMAT, Toulouse INP, Université Toulouse 3 Paul Sabatier, CNRS, Université de Toulouse, 4 Allée Emile Monso, BP44362, CEDEX 4, 31030 Toulouse, France; 2 LCC, UPR 8241 CNRS, Université de Toulouse, 205 Route de Narbonne, CEDEX 4, 31077 Toulouse, France; 3 Université de Bordeaux, CNRS, Bordeaux INP, I2M, UMR 5295, 33400 Talence, France; 4 Arts et Metiers Institute of Technology, CNRS, Bordeaux INP, Hesam Universite, I2M, UMR 5295, 33400 Talence, France; 5 CIRIMAT, Toulouse INP, Université Toulouse 3 Paul Sabatier, CNRS, Université de Toulouse, 118 Route de Narbonne, CEDEX 9, 31062 Toulouse, France

**Keywords:** amorphous calcium phosphate, porosity, bone scaffolds, cold sintering

## Abstract

A large amount of research in orthopedic and maxillofacial domains is dedicated to the development of bioactive 3D scaffolds. This includes the search for highly resorbable compounds, capable of triggering cell activity and favoring bone regeneration. Considering the phosphocalcic nature of bone mineral, these aims can be achieved by the choice of amorphous calcium phosphates (ACPs). Because of their metastable property, these compounds are however to-date seldom used in bulk form. In this work, we used a non-conventional “cold sintering” approach based on ultrafast low-pressure RT compaction to successfully consolidate ACP pellets while preserving their amorphous nature (XRD). Complementary spectroscopic analyses (FTIR, Raman, solid-state NMR) and thermal analyses showed that the starting powder underwent slight physicochemical modifications, with a partial loss of water and local change in the HPO_4_^2-^ ion environment. The creation of an open porous structure, which is especially adapted for non-load bearing bone defects, was also observed. Moreover, the pellets obtained exhibited sufficient mechanical resistance allowing for manipulation, surgical placement and eventual cutting/reshaping in the operation room. Three-dimensional porous scaffolds of cold-sintered reactive ACP, fabricated through this low-energy, ultrafast consolidation process, show promise toward the development of highly bioactive and tailorable biomaterials for bone regeneration, also permitting combinations with various thermosensitive drugs.

## 1. Introduction

In vertebrates, the endoskeleton ensures the protection of internal organs while allowing movement and the body’s mechanical stability. At a lower scale, the bony matrix also hosts the development of blood cells in the bone marrow and takes part in homeostasis processes. In this view, skeletal restoration, in the event of traumas and bone diseases, is a major surgical challenge worldwide and is a key focus in orthopedic, dental and cranial surgeries. This is all the more crucial taking into account the global ageing of populations, generating an increasing number of surgical operations and bone-related pathologies such as osteoporosis, pseudo-gout or arthrosis. Although autografts have long been considered as the gold standard for bone repair, the limitation of available graft tissue along with the necessity for a secondary surgical site have exponentially pushed forward the development of synthetic bone substitutes.

The development of synthetic bone-related materials sometimes requires to depart from the original chemistry and/or (micro)structure of native bone. Since the biomaterials need to exhibit some degree of cohesion and mechanical properties adapted to their clinical use, the technical specifications for synthetic bone substitutes have to be take into account surgical handling and post-operative constraints. With this view, 3D approaches are being investigated to provide bone substitutes with enhanced mechanical properties via sintering (e.g., [1]). In order to provide bioactivity features, bone graft materials are increasingly studied to enhance their osteointegration, for example, through coatings intended to mimic the native mineral phase to further enhance the cell colonization and activity [2]. Thus, nowadays, a large amount of research is dedicated to the search for ever-more bioactive compounds, not only capable of physically occupying the bone defects but also triggering cell activity for promoting bone regeneration [3].

Taking into account the chemical composition of bone, constituted of about 70 wt.% calcium phosphate (CaP)—a nanocrystalline, nonstoichiometric, apatitic phase—many studies are being run on the setup of reactive CaPs as bone substitutes [4]. Such biomaterials are indeed particularly suitable for the replacement or repair of bone defects in non-load bearing sites. The reactivity may originate from the chemical nature of the phase(s) involved that are thermodynamically metastable in vivo. It can also be modulated by physical parameters such as the extent of surface in contact with body fluids, e.g., via a more or less porous matrix. Even for non-load bearing sites, it is important to prepare scaffolds with sufficient mechanical resistance allowing for manipulation, surgical placement and eventual cutting/reshaping in the operation room. 

To achieve this goal, samples initially prepared as powders need to be consolidated into cohesive 3D scaffolds which, for ceramic-based systems such as CaPs, require some densification process. The sintering of CaP phases such as stoichiometric hydroxyapatite (HA) or β-tricalcium phosphate (β-TCP) is a well-known process, run at high temperature (typically > 900 °C), allowing to obtain highly densified constructs [5,6]. These bioceramics are widely commercialized, for example, in the form of biphasic HA/β-TCP granules, and prove especially helpful where low resorption is needed. However, these systems exhibit a very limited bioactivity, low surface areas and poor surface reactivity with low affinity for drugs/active (bio)molecules in comparison with their less crystallized or amorphous counterparts [7,8]. Moreover, it is not possible to use such “conventional” high-temperature sintering approaches for the consolidation of metastable phases such as biomimetic apatites, octacalcium phosphates or amorphous calcium phosphates, among other phases that are hydrated and often involve nanosized constitutive particles. 

In the case of such thermally unstable compounds, there is a need to develop alternative strategies. The so-called “cold sintering” methodologies are, in this view, pertinent unconventional ways to provide consolidation while limiting the alteration of the starting powder, e.g., in terms of particle size, nature of the crystallographic phase and hydration state. Cold sintering may be seen as a group of methods that carry out a densification/consolidation of the powder precursor at a rather “low” temperature (for ceramic compounds), typically lower than 300 °C. It encompasses several types of approaches, with or without the application of mechanical pressure, with or without (limited) heating, and for various treatment durations. The consolidation of biomimetic nanocrystalline apatites has been shown, since 2006 [9,10], to be possible by Spark Plasma Sintering (SPS) at a “low” temperature (e.g., 13 min at 150 °C under 100 MPa) over few minutes, while preserving the hydrated character of the apatite nanocrystals and their reactive amorphous surface layer. This approach was later extended to amorphous CaPs [11,12] which lies among the most resorbable, metastable and bioactive CaPs phases, thus with high expectations for further biological osteointegration, drug association and bone regeneration [7]. However, the SPS approach generally leads to crystallization into poorly crystallized apatite. The possibility to retain a fully amorphous compound after low temperature SPS processing was however shown by co-doping the ACP phase with Mg^2+^ and CO_3_^2−^ ions, known as apatite growth inhibitors. Apart from SPS—exploiting the application of pulsed electrical current—other methodologies have also been explored. Compression heating was, for example, reported to achieve the consolidation of nanocrystalline apatite under higher applied mechanical pressures (e.g., at 200 °C under 500 MPa [13,14]). Whether by compression heating or by SPS, such a “cold” sintering mechanism is thought to be achievable for nanocrystalline biomimetic apatites because of the presence of an amorphous ionic hydrated layer on the nanocrystals, where the ions and water molecules are highly mobile and may diffuse to create intercrystalline bonding even without high thermal activation [10,15]. Indeed, on biomimetic apatites or ACPs, the constitutive water already present on the particles is likely to generate, during the consolidation process, a transient liquid phase favoring the diffusion of ions and thus the binding of the grains. In this view, the cold sintering of such compounds does not necessitate the addition of an external liquid phase, unlike what was later proposed in the literature for non-hydrated compounds [16]. Recently, the addition of an acidic liquid phase to already hydrated apatite (obtained via the processing of mussel shells) was nonetheless tested and shown to further increase, if needed by the intended clinical use, the densification rate of cold-sintered nanocrystalline apatites [17]. In ACPs, although there is, to this date, no real consensus on a possible mechanism of consolidation, the presence of a significant amount of water molecules allied with the low structural order of the amorphous phase are also susceptible to explain the possible sinterability even at low temperatures. Attempts to consolidate ACPs at room temperature by relying on high applied mechanical pressure (e.g., up to 1.5 GPa) have been reported [18]. In this case, very high densification rates were accessed (e.g., 98.4%). However, in the field of bioceramics, the presence of residual porosity—and thus only partial densification—may be seen as an asset for some clinical applications as in non-load-bearing sites, to promote cell activity, bone repair and microvascularization, and also in view of the potential release of added bioactive agents such as bioactive ions or (bio)molecules/drugs. 

In this context, we explored in this contribution the consolidation of a reactive ACP phase prepared by spray drying, by ultrafast compression (thousand-fold faster than usually encounter in the literature) under low mechanical pressure (200 MPa and under) and at room temperature. To the best of the authors’ knowledge, this paper reports the first fabrication of a highly porous non-brittle ACP biomaterial through such a compression technique and maintenance of its amorphous character. Macroscopic properties such as mechanical resistance and porous architecture were considered with the final goal to fabricate 3D biomaterials that could be handled and cut and propitious for boosting bone tissue repair. From a more microscopic viewpoint, chemical and structural modifications upon processing were also explored, using several complementary techniques, including solid-state NMR, FTIR and Raman spectroscopies and thermal analyses. By assessing the specific physicochemical features of such 3D scaffolds of reactive ACP and a low-energy, ultrafast consolidation process, this study paves the way to the development of new highly active and porous biomaterials for bone regeneration applications.

## 2. Materials and Methods

### 2.1. Reagents

Phosphoric acid, PA (H_3_PO_4_ 85%), and monohydrate calcium acetate, CA (Ca(CH_3_COO)_2_, H_2_O), were purchased from Sigma Aldrich (Darmstadt, Germany).

### 2.2. Sample Synthesis and Physicochemical Characterization

#### 2.2.1. ACP Synthesis and Spray Drying

ACP powder was obtained using our previously reported spray drying protocol [19]. Briefly, calcium and phosphate precursors (5 g CA and 1.5 mL PA) were dissolved in 1 liter of deionized water under vigorous stirring. The resulting, highly diluted solution was then spray-dried using a B 290 mini spray dryer from BUCHI (Büchi Labortechnik AG, Flawil, Switzerland) with an inset temperature of 160 °C, an air flow of 414 L·h^−1^ and a liquid flow of 0.3 L·h^−1^. In this process, the droplets formed during spray drying were used as microreactors, in which the solvent evaporation induced the in situ ACP precipitation. The Ca/P molar ratio of the initial solution was intentionally set to 1.3 to minimize the presence of calcium acetate in excess in the final product, according to our reference work mentioned above.

#### 2.2.2. Compression Process

**Figure 1:**
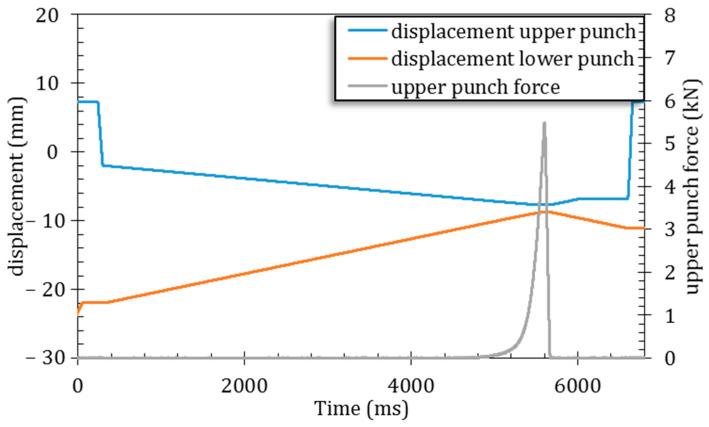
Consolidation of ACP powder by symmetrical compression: example of displacement of the punches and the resulting force in the case of the 200 MPa sample.

Consolidation of the prepared ACP powder was carried out with a pharmaceutical-grade compaction simulator Styl’One Evolution (Medelpharm, Beynost, France), using EuroB Flat-faced punches with a diameter of 6 mm. The die was manually filled with 50 mg and externally lubricated with magnesium stearate (Ligamed MF-2-V, Peter Greven, Bad Münstereifel, Germany). Experiments were performed by symmetrically applying a mechanical pressure chosen in the range 25–200 MPa, at room temperature and with a dwell time of 20 ms. An example of the displacement of the two punches and the resulting force is given in Figure 1. For most of the compression cycle, no force is transmitted to the upper punch. In fact, from 0 to ~4700 ms, the rearrangement of the particles is expected to be the primary mechanism involved in the compaction process. Additionally, crumbling may also occur during this phase as the micrometric-sized grains of ACP are not expected to exhibit strong cohesion. The duration for which a force is transmitted to the upper punch corresponds to 1000 ms (from 4700 to 5700 ms). However, for convenience, only the dwell time (period during which the force is at its set value) of 20 ms is presented in this study. It may be highlighted that we purposely chose to apply pressure over a very short timeframe (20 ms dwell time) in order to avoid undesirable relaxation phenomena. Preliminary tests performed over 60 s indeed showed a progressive accommodation of the sample to the mechanical strain, with a relaxation observed on the force-versus-time curve (Figure S1 in Supplementary Information).

#### 2.2.3. Tensile Strength

The tensile strength of the compressed pellets was evaluated by the diametral compression test (also called the Brazilian test) with a TA.HDplus apparatus (Stable microsystems, Surrey, UK). The tests were run at a speed of 0.1 mm·s^−1^ over a path of 0.5 mm. The tensile strength was calculated using the following Equation (1):

(1) $$\sigma =\frac{2\cdot F}{\pi \cdot D\cdot t}$$

where *F* is the maximum force reached during the test, *D* is the diameter of the pellet and *t* its thickness [20]. The σ value are presented as the mean ± standard deviation of the mean. Statistical differences between mean values were assessed using one-way analysis of variance (ANOVA).

#### 2.2.4. Porosity and Specific Surface Area

The extent of porosity “*p*” was evaluated from density measurements as follows:

(2) $$p=1-\frac{\rho_{sample}}{\rho_{pycno}}$$

where *ρ_sample_* was measured from the mass and volume of the sample (either as starting powder or after consolidation) and *ρ_pycno_* was determined using a helium pycnometer (AccuPyc 1330, Micromeritics, Norcross, GA, USA) with 20 purges and 20 runs. The experiments were run at least in triplicate and given as the mean ± standard deviation.

The N_2_ absorption–desorption isotherm (Tristar II 3020 Micromeritics, Norcross, GA, USA) was used to determine the specific surface area by the Brunauer-Emmet-Teller (BET) method; and the pore size was calculated using the Barret–Joyner–Halenda (BJH) model. The samples were degassed at 80 °C for 5 h.

#### 2.2.5. Fourier-Transform Infrared (FTIR) Spectroscopy

FTIR spectroscopic analyses were run in transmission mode using a FT/IR Nicolet 5700 spectrometer (Thermo, Loos, France), in the 400–4000 cm^−1^ wavenumber range with a 2 cm^−1^ resolution and 64 accumulations. Approximately 5 mg of the consolidated ACP were gently grinded with 300 mg of KBr and pressed under 7 T for 30 s. The spectra were normalized using the ν_3_PO_4_ 1070 cm^−1^ band.

#### 2.2.6. Raman Microspectroscopy

Raman analyses were carried out using a confocal RAMAN Labram HR 800 Horiba Yvon Jobin microscope. The samples were exposed to continuous laser radiation supplied by a He/Ne laser at λ = 633 nm with a power of 1 mW for single point analysis. The samples were placed under an Olympus BX 41 microscope and focused under a ×100 objective with a numerical aperture of 0.90.

A silicon standard enabled the equipment to be frequency-calibrated using the 1st order line of silicon at 520.7 cm^−1^ with an accuracy of ±1 cm^−1^.

Each spectrum was acquired using a 600 tr/mm grating with a spectral resolution of 1 cm^−1^ and collected with a quantum well detector cooled to −60 °C by the double Pelletier effect (CCD Synapse). The acquisition time was set to 60 s, with 3 accumulations. Data processing was performed using the Labspec 6 software (v 6.6.1.11).

#### 2.2.7. Solid-State NMR

NMR experiments were recorded on a Bruker Avance 400 III HD spectrometer operating at a magnetic field of 9.4 T. The samples were packed into 1.3 mm zirconia rotors and spun at 58 kHz for ^1^H and ^31^P experiments, at room temperature. ^1^H MAS analyses were performed with the DEPTH pulse sequence using a recycle delay of 10 s. The ^1^H-^31^P-^1^H Flip-Back CP (FBCP) NMR method (^1^H→^31^P→^1^H) was used with a first CP transfer of 5 ms and a second CP contact time of 0.2 s. ^31^P MAS spectra were recorded with a recycle delay of 600 s.

#### 2.2.8. X-ray Diffraction (XRD)

XRD analyses were carried out with a D8-Advance (BRUKER, Billerica, MA, USA) diffractometer using a Cu anticathode (λ = 1.54184 Å) in the 2θ range 10–90° with a step of 0.02° and an accumulation time of 1 s per step. No background correction was applied to avoid any loss of information.

#### 2.2.9. Thermal Analyses

The thermal behavior of the starting powder and compressed pellets was investigated using a SETARAM (Setsys Evolution System, Caluire-et-Cuire, France) TGA/DTA apparatus from 25 to 1000 °C at 5 °C/min under air flow (20 mL/min) preceded by a plateau at 25 °C for 30 min for preliminary equilibration. 

#### 2.2.10. Scanning Electron Microscopy (SEM)

The microstructure of the pellets was analyzed by scanning electron microscopy (SEM) using a FEI Quanta450 microscope (Midland, ON, Canada). The samples, non-conductive and metastable, were observed at low vacuum (130 Pa of water vapor), with an electron beam acceleration of 10 kV and an intensity of 2.5 pA. The cross section of the pellets was observed after diametral compression tests, with no metallization of the sample.

## 3. Results and Discussion

Among calcium phosphates, amorphous compounds (ACPs) are particularly relevant for the fabrication of reactive and resorbable bone substitutes, taking into account their non-crystalline nature and related thermodynamic metastability, potentially relevant to produce readily resorbable scaffolds. In this work, the ACP powder was prepared (as detailed in our previous paper [19]) by spray drying to generate spherical particles with a very short maturation time, thus yielding highly reactive ACP. In this synthesis route, the solution droplets are used as microreactors for the in situ precipitation of ACP. The Ca/P molar ratio was purposely selected at 1.3—thus lower than the traditional 1.5 ratio encountered for most ACPs in the literature. It was indeed shown that this molar ratio allowed obtaining pure reactive ACP particles while drastically limiting by-products such as crystalline calcium acetate. 

**Figure 2:**
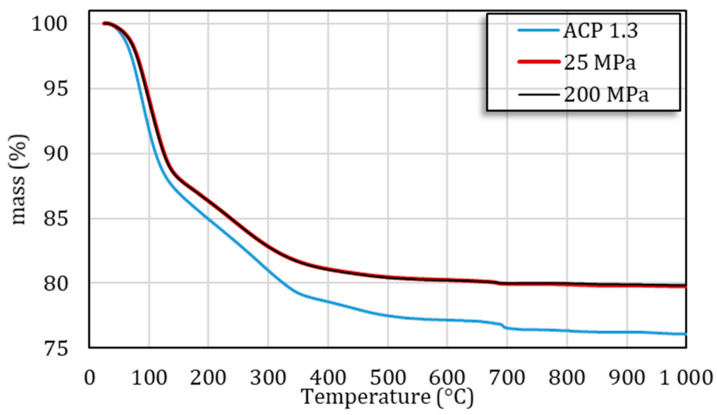
Thermal analysis of the ACP starting powder and the consolidated pellets obtained under 25 and 200 MPa.

The overall chemical composition of this ACP sample can be approximated to Ca_9_(PO_4_)_4_(HPO_4_)_3_. nH_2_O. The thermogravimetry data (Figure 2) allowed estimating the amount of water to *n* ≈ 15.5 (with a residue of amorphous calcium acetate estimated to 0.33 molecule per ACP cluster), with 62% poorly bound (physisorbed) water and 38% “structural water” as assessed by the two significant heat effects observed in the ranges 20–200 °C and 200–340 °C, respectively (Figure S2a). This structural water has been previously hypothesized, in our earlier study, as pertaining to a hydrated layer surrounding constitutive ACP clusters. Rewriting this chemical composition by revealing the amorphous tricalcium phosphate moiety “Ca_3_(PO_4_)_2_” leads to [Ca_3_(PO_4_)_2_]_2_·[CaHPO_4_]_3_·15.5 H_2_O; and this chemical formula corresponds to a Ca/P ratio of ~1.29. Taking into account all experimental uncertainties, all these findings fit reasonably well with the initially anticipated Ca/P molar ratio of 1.3 mentioned above.

**Figure 3:**
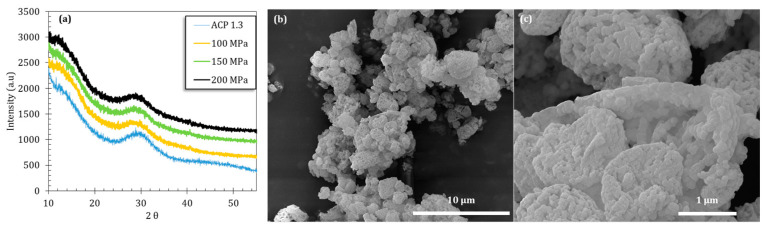
(**a**) XRD patterns of the initial powder and pellets made at 100, 150 and 200 MPa. The SEM images (**b**,**c**) present the overall powder and a magnification on several grains.

The amorphous character of the obtained powder after spray drying was confirmed by XRD analysis (Figure 3a). The particles, observed by SEM, were found to exhibit a globular shape and a high degree of porosity, with a mean size of around 2.4 µm (laser diffraction analysis), and were themselves constituted of nanoparticles of ca. 100 nm (Figure 3b,c). Analysis by SAXS presented in our previous study [19] also revealed the existence of ca. 2 nm clusters at a smaller scale. Thus, the particles constituting the ACP starting powder used in this work exhibit a multiscale structure that may take part in its overall reactivity/consolidation behavior.

This spray-dried powder exhibits a high degree of porosity, reaching 99% as determined by He pycnometry, and the BET specific surface area was evaluated to be 20.71 ± 0.09 m^2^/g. 

**Figure 4:**
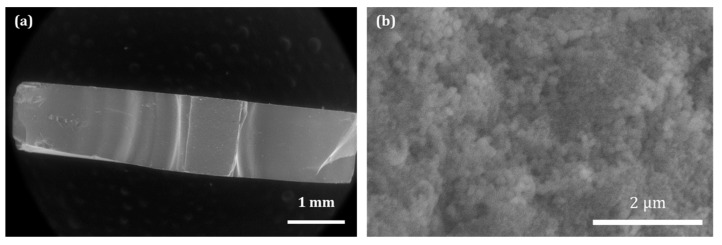
SEM images: (**a**) corresponding to a 100 MPa pellet after Brazilian test; (**b**) magnification of the center of a pellet.

This peculiar ACP powder was then used, in the present study, to obtain 3D biomaterials by ultrafast symmetrical die compression. Tests were carried out under various applied pressures ranging from 25 to 200 MPa, to inspect the effect of the mechanical stress on the ACP particles. In all the cases tested, 3D pieces were successfully produced (6 mm diameter × 1 mm height for a final mass of 47 ± 3 mg). A characteristic SEM picture of a pellet section is presented in Figure 4 (after diametral compression test). No noticeable difference was observed using SEM among the samples (Figure S3) obtained at different pressures: in all cases, the consolidation of the powder resulted in a rather homogeneous network of 100 nm nanoparticles (NPs) and the disappearance of the micrometric particles, visible in the starting spray-dried powder (Figure 4a). These findings are indicative of the poor mechanical properties of such micron-sized particles, while the 100 nm NPs remain essentially undistorted. The porosity of the pellet is observable in Figure 4b and is induced by the rearrangement of the 100 nm nanoparticles.

**Figure 5:**
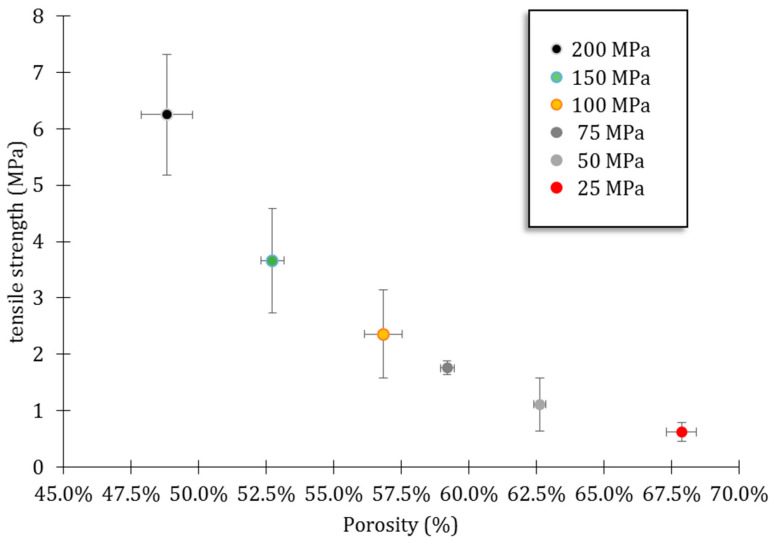
Tensile strength as a function of porosity for different compaction pressures applied.

The variation of the tensile strength versus the total porosity, as drawn from He pycnometry and N_2_ absorption–desorption, is reported in Figure 5. For consolidation pressures from 25 to 200 MPa, the tensile strength ranged from 0.63 ± 0.17 to 6.25 ± 1.07 MPa for pore volumes from 67.8 ± 0.5 to 48.8 ± 1%. The results show the expected behavior of increased densification and improved tensile strength when increasing the applied pressure on the powder. These two phenomena are correlated, as increasing the pressure brings the small 100 nm particles closer to each other, thereby increasing the density of the scaffold. In turn, the additional particle/particle interactions increase its mechanical properties. Moreover, the increase in the tensile strength for every 50 MPa step is significant (*p* < 0.05) while the decrease in the porosity is highly significant (*p* < 0.01). According to Wagoner et al. [21], all the pellets are in the target range of porosity (e.g., 30–90%) for suitable cancellous bone replacement. We arbitrarily established the tensile strength threshold at 2 MPa to ensure sufficient mechanical properties to withstand manipulations. This threshold is of the order of magnitude of the mechanical threshold applied for pharmaceutical tablets to ensure commercial manufacture and subsequent distribution [22]. Importantly, the uncertainty on the tensile strength measure increases with the applied pressure (decreasing porosity). This may be related to the partial lamination of the pellet during the test, resulting in an under-evaluation of the strength and a wider range of measurements, even though the pellet did not present visible defects after die unmolding. Note that the pellets that underwent lamination during the Brazilian test and did not break properly through the center of the pellet were excluded from the calculation of the average and the standard deviation (this concerns 2 pellets over 10 for 200 MPa and 1 over 10 for 100 MPa).

**Figure 6:**
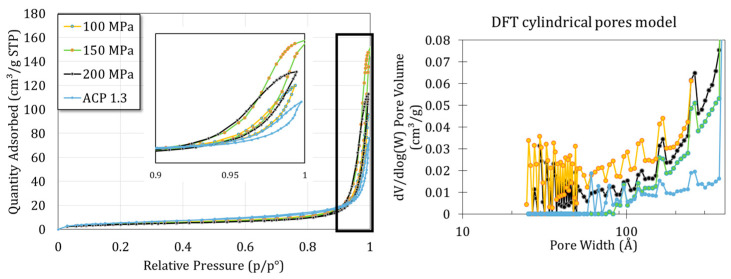
N_2_ absorption–desorption isotherm and DFT model for the powder and the pellet obtained at 100, 150 and 200 MPa.

Figure 6 shows the H_2_ adsorption–desorption isotherms of the samples obtained at 100, 150 and 200 MPa and for the starting ACP powder. A mix between type II (macroporous) and IV (mesoporous) behaviors can be identified with a hysteresis type H1. Such types II and IV isotherms were indeed expected with regards to the SEM images evidencing the presence of interparticle voids. A cylindrical pore model was implemented, taking into account the mean size and spherical shape of the nanoparticles. This model then suggested the presence of mesopores of about tens of nanometers, which seems consistent with a compact rearrangement of 100 nm nanoparticles.

Finally, the specific surface area was determined using the BET method. Results show a slight decrease in the specific surface area with the increase in applied pressure, from 20.71 ± 0.09 m^2^/g for the initial powder to 17.67 ± 0.09, 16.41 ± 0.10 and 15.25 ± 0.08 for the 100, 150 and 200 MPa pellets, respectively (*p* < 0.01). These data point out a successful densification of the ACP powder.

**Figure 7:**
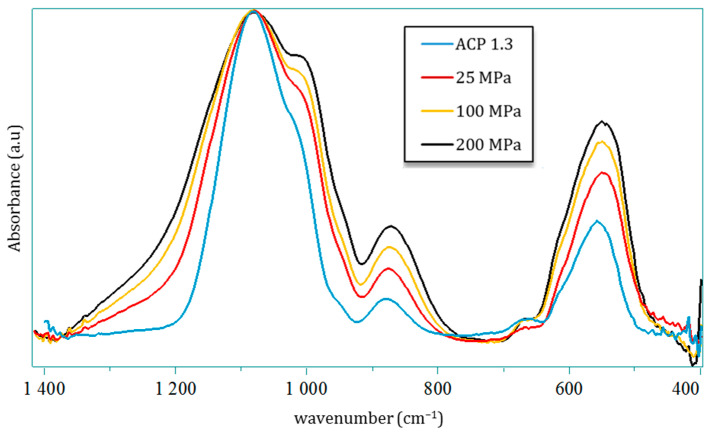
FTIR spectra in the [1400, 400 cm^−1^] windows of the powder and the pellet obtained at 25, 100 and 200 MPa and normalized with the ν_3_(PO_4_) at 1070 cm^−1^.

After compression, all samples were found to remain amorphous on XRD, and Figure 3 displays the typical examples of the ACP powder pressed under 100, 150 or 200 MPa in comparison to the starting powder. In all cases, the broad diffraction halo characteristic of amorphous compounds can be observed with no peak sharpening nor shift of the halo maxima. In order to further investigate possible changes undergone by the precursor ACP powder, FTIR analyses (in transmission mode) were run. As pointed out in Figure 7, despite global spectra characteristic of amorphous calcium phosphate, modifications may be identified in the ν_3_(PO_4_) and ν_4_ (PO_4_) domains. These bands are by definition complex superimpositions of several spectral contributions [23]. Several trends may be drawn on the global shape of the ν_3_(PO_4_) band upon varying the mechanical pressure with, on one hand, a broadening effect especially on the high frequency side and, on the other hand, an increase in relative intensities of two sub-components at 1010 and 1080 cm^−1^. The ν_4_ domain shows only little modification, except for a more prominent shoulder at 604 cm^−1^. These findings may probably be related to observations made in the literature on CaP compounds, for which the relative intensity of ν_3_ and ν_4_(PO_4_) sub-components was found to be dependent on compositional/structural features of the samples. For example, in bone/enamel, the intensity of the 1030 cm^−1^ contribution was shown to be especially prominent for mature samples, while a shoulder at ca. 1110 cm^−1^ or 1125 cm^−1^ is more visible for immature samples. A progressive inversion of low wavenumber/high wavenumber relative intensities in the ν_3_(PO_4_) domain, close to our findings, has for example been observed by investigating dental enamel from the immature (outer) to mature (inner) tissue [24]. Also, in the follow-up of ACP-to-apatite transformation [25], the increase in a contribution at 999 cm^−1^ and a decreased intensity at 1055 cm^−1^ were reported along the progression of the conversion. It may however be noted that such literature reports on amorphous or metastable CaPs were not obtained by application of a mechanical pressure nor through a consolidation process. The only FTIR studies reporting, to our knowledge, the influence of mechanical pressure were carried out on highly crystallized CaP phases [26,27,28,29,30]. Although spectral modifications were seen in these cases in the ν_3/_ν_4_(PO_4_) domains (in terms of relative intensities, position and bands widening), they were found to be reversible upon pressure release, which does not seem to be the case in our present work on ACP. This could tentatively be explained by the lack of long-range order found in ACP or immature/mestastable CaPs like nanocrystalline apatite, compared to highly crystalline phases.

Our observations, altogether, suggest that our ACP sample—while remaining amorphous on XRD—underwent a progressive structuration upon mechanical compression. Beside the modification in relative intensities between low and high components in this spectral domain, we also evidenced a shift of the local maximum toward lower wavenumbers of the 1000–1010 cm^−1^ contribution (from 1010 cm^−1^ for the initial powder down to 997 cm^−1^ for 200 MPa) upon compression. However, this is probably not assignable to a red shift tendency of a band, as one might expect at first sight. Instead, this effect is likely attributable to the progressive fading of underlying contributions located at slightly higher wavenumbers [31] or vice versa, an increase in a contribution at lower wavenumber. 

**Figure 8:**
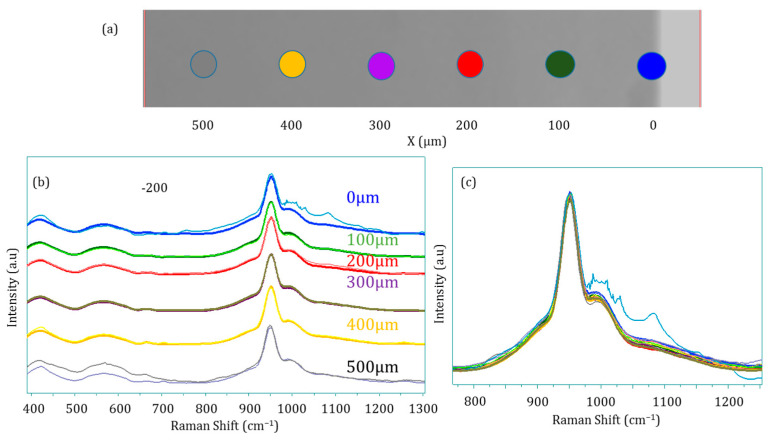
Raman multi-point spectral analyses along the compression axis: (**a**) location of the spots, (**b**) corresponding Raman spectra (light colors refer to 100 MPa and dark colors to 200 MPa) and (**c**) superimposition of all spectra in the 800–1200 cm^−1^ range.

The above findings unveiled a structural modification of ACP particles upon consolidation, all the more prominent that the applied mechanical pressure is high. These conclusions were drawn from vibrational spectroscopy, which probes the local environment of (phosphate) ionic species, while the global XRD signature still points to amorphous compounds. Therefore, the modification of the ACP structuration upon compression likely arises from short-distance changes in the close vicinity of phosphate groups. To investigate if these modifications occur in the depth of the pellet, Raman multi-point spectra were performed on a pellet cross section after the Brazilian test. A spectrum was recorded every 100 µm from one external face of the pellet down to 500 µm in depth (corresponding to half the thickness of the pellet), thus along the compression axis. The spots corresponding to the Raman multi-point acquisition of the 200 MPa pellet are presented in Figure 8a (dark curves), while the spectra obtained are shown in Figure 8b. This also includes the spectra of the 100 MPa pellet obtained in the same manner (lighter colors). Finally, Figure 8c presents the superimposition of all the spectra in the 800–1200 cm^−1^ range to highlight possible differences. The main and most obvious difference concerns the spectrum obtained on the surface of the pellets (in light blue color), as it is the only one that exhibits the characteristic bands attributed to magnesium stearate used as the lubricant in this experiment. Beside this difference, no significant alteration of the spectra can be observed in the depth of the pellet, indicating the homogeneity of the material along the compression axis. Interestingly and unlike the FTIR spectra, no detectable differences are observed between the 100 and 200 MPa spectra. Taking into account that Raman spectroscopy is poorly sensitive to the HPO_4_^2−^ ions and that the results are given for micronic volume size unlike in FTIR where it is carried out on a larger volume, the differences observed previously in FTIR can be potentially attributed to the contribution of HPO_4_^2−^ in the ν_3_ and ν_4_ PO_4_ domains [32].

One possible evolution of such an ACP could be linked to a modification of its hydration state [33]. To explore this hypothesis, thermal analyses were carried out, through TGA measurements. The results (Figure 2) indicate that compressed ACP exhibits a lower overall weight loss (typically in the 25–340 °C temperature range) than the initial powder, indicating that the compression process itself was responsible for a partial loss of water. No significant difference was observed between the samples consolidated at 25 and 200 MPa; in both cases, about 23% of the total amount of water was lost during processing, with a more prominent loss (about twice as much) of the “structural” water compared to free water. The partial loss of water and local variations affecting the hydrated domains inspire some similarity with the low-temperature consolidation/cold sintering of biomimetic apatites [9,10], which was explained on the basis of a high labile character of ions within the hydrated surface layers of adjacent nanocrystals, thus favoring their diffusion upon consolidation to allow densification even at low temperatures. For temperatures higher than 340 °C and up to 600 °C, an additional weight loss of ~2 wt.% is detected on the thermograms and can be attributed to the loss of water following the condensation of HPO_4_^2−^ ions into pyrophosphate ions P_2_O_7_^4−^. This is consistent with the initial amount of HPO_4_^2−^ ions. The presence of β-Ca_2_P_2_O_7_ was indeed detected beside β-TCP by Raman analysis carried out after TG analysis up to 1000 °C (Figure S2b). Note that a third weight loss (~0.4–0.5 wt.%) may be seen around 700 °C for the consolidated ACP pellet, which can be attributed to the loss of CO_2_ arising from the minor acetate residue [34]. In comparison, the starting powder exhibited a weight loss of 1 wt.% at 700 °C. This decrease upon compaction thus indicates that part of the residual acetate was lost during the compaction process itself, which may presumably be related to its rather volatile character.

**Figure 9:**
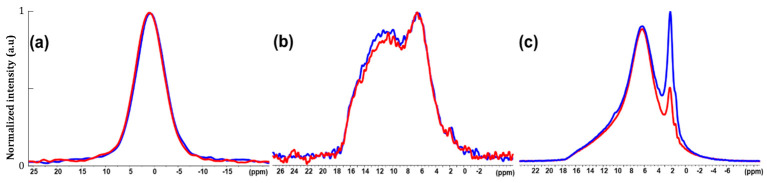
(**a**) ^31^P, (**b**) ^1^H-^31^P-^1^H FBCP and (**c**) ^1^H MAS NMR of ACP 1.3 (blue) and 200 MPa (red).

At this point, solid-state NMR analyses were run, before and after consolidation (at 200 MPa as illustrative example), to complement the compositional and structural investigations. ^31^P CP MAS NMR spectra (Figure 9a) exhibit no significant differences between the powder and the 200 MPa compact, suggesting that the average environment of the phosphorus nuclei (i.e., within PO_4_ and HPO_4_) seems essentially preserved. This is coherent with both the Raman and XRD analyses presented above. Nevertheless, in comparison with the initial powder, the ^1^H-^31^P-^1^H FBCP spectrum of the consolidated ACP (Figure 9b) shows a slight relative decrease in magnetization transfer between the P and H nuclei in the [10–16 ppm] range compared with the [6–7 ppm] H area corresponding to the HPO_4_^2-^ ions and structural water, respectively. This indicates a closer proximity between the average P nuclei and the H nuclei of the structural water than H nuclei of the HPO_4_^2-^ ions; this is either related to an increase in the average P-H distances in the HPO_4_^2-^ ions or related to a decrease in the relative amount of HPO_4_^2−^ ions in the compact. This effect is however minor and therefore probably only concerns few ions. ^1^H MAS NMR (Figure 9c) presents an important decrease in the 2.1 ppm peak attributed to the CH_3_COO^−^ ion (as a remaining impurity after spray drying) in the pellet in comparison with the powder, accompanied by a more discrete decrease in the 6 ppm peak (structural water). This result indicates a partial loss of the acetate during the compaction, which is in full agreement with the TG analyses. This observation can likely be related to the low evaporation temperature of acetate and the fact that the compaction can lead to a local increase in the temperature at the contact point(s) between two grains/nanoparticles [35,36]. Consequently, like FTIR, solid-state NMR data suggest only few modifications of the ACP compound upon such fast consolidation under pressure, which presumably affects mostly some elements constituting the hydrated domains of the particles, while the “core” of the particles seems relatively unchanged, which could also explain the XRD outcomes.

## 4. Conclusions

All of the above results point out the successful consolidation, by cold sintering at RT under ultrafast pressing, of an ACP powder corresponding to the overall composition [Ca_3_(PO_4_)_2_]_2_·[CaHPO_4_]_3_·15.5H_2_O (0.33 acetate) obtained by spray drying. The effect of the applied mechanical pressure on the extent of porosity and related mechanical properties was clearly evidenced. For every pressure tested, SEM observations showed good spatial homogeneity of the pellet, without inner defects (cracks or observable density inhomogeneity), traces of lamination or obvious compaction-related heterogeneity at the die output. 

Comparison of physicochemical features between the consolidated pellets and the initial powder was carried out on the basis of various complementary analyses. Importantly, the amorphous character of this ACP compound was shown to be preserved after such a consolidation process, as confirmed by XRD, despite a significant loss of water (and acetate minor impurity) inherent to the sintering mechanism. In contrast, spectroscopic techniques—allowing us to probe the local environment of constitutive ions/molecules—unveiled some degree of modification at a more discrete scale. Indeed, solid-state NMR results suggested some changes in the hydrated domains while showing a relative stability of the average phosphorus nuclei environment. Vibrational spectroscopy through the analysis of the ν_3_(PO_4_) and ν_4_(PO_4_) domains by FTIR pointed out slight modifications related to the applied pressure, that remind the evolution of immature hydrated CaPs upon ageing. While the amorphous nature of the pellet is undeniable, the constitutive particles undergo some local modifications during the compaction. The underlying mechanism at play in this ultrafast pressure-assisted cold sintering approach is not yet fully elucidated. 

Due to the low pressure applied on the powder (up to 200 MPa) compared to the high pressure-based sintering literature on CaPs (pressures of the order of GPa), the ACP pellet presents a high porosity with mesopores and mechanical properties allowing the pellet to be easily handled and cut (e.g., in the surgical room). Also, the “still amorphous” character of the pellets is expected to be beneficial in view of the fast evolution/biodegradation in vivo. Moreover, thanks to the low temperatures involved here, the addition of drugs can also be envisioned to provide additional functionalities, e.g., enhancing the bioactivity of the bone-filling material, by exploiting the porous network that can be tuned by the applied pressure. Finally, the preparation of the starting powder by another soft chemistry route, namely spray-drying, also allows combining thermosensitive (bio)molecules/drugs in the initial solution, thus providing another path for modulating the therapeutic properties of the final implanted scaffolds.

## Data Availability

Data are available by contacting the corresponding author.

## Supplementary Materials

The following supporting information can be downloaded at: https://www.mdpi.com/article/10.3390/nano14020152/s1, Figure S1: Compression test at 200 MPa with a 60 s cycle; Figure S2: (a) DTA analyses of the powder and the 25 MPa and 200 MPa pellets and (b) Raman spectra of the 25 MPa pellet before and after TGA/DTA analysis; Figure S3: SEM pictures of 100, 150 and 200 MPa samples in 130 Pa and vacuum.
