# Supplementary material for: Consolidation of Spray-Dried Amorphous Calcium Phosphate by Ultrafast Compression: Chemical and Structural Overview
Source: Nanomaterials (Basel). 2024 Jan 10;14(2):152. doi: 10.3390/nano14020152 (PMC10819566; doi:10.3390/nano14020152)
Supplement: Supplementary file 1 [file nanomaterials-14-00152-s001.zip › nanomaterials-2781704-supplementary.pdf]

Consolidation of Spray-Dried Amorphous Calcium Phosphate by Ultrafast Compression: Chemical and Structural Overview

Sylvain Le Grill [1,*], Christophe Drouet [1], Olivier Marsan [1], Yannick Coppel [2], Vincent Mazel [3,4], Marie-Claire Barthelemy [5] and Fabien Brouillet [5]

[1] CIRIMAT, Toulouse INP, Université Toulouse 3 Paul Sabatier, CNRS, Université de Toulouse, 4 Allée Emile Monso, BP44362, CEDEX 4, 31030 Toulouse, France
[2] LCC, UPR 8241 CNRS, Université de Toulouse, 205 Route de Narbonne, CEDEX 4, 31077 Toulouse, France
[3] Université de Bordeaux, CNRS, Bordeaux INP, I2M, UMR 5295, 33400 Talence, France
[4] Arts et Metiers Institute of Technology, CNRS, Bordeaux INP, Hesam Universite, I2M, UMR 5295, 33400 Talence, France
[5] CIRIMAT, Toulouse INP, Université Toulouse 3 Paul Sabatier, CNRS, Université de Toulouse, 118 Route de Narbonne, CEDEX 9, 31062 Toulouse, France
* Correspondence: sylvainlegrill@gmail.com

Supplementary information

Style One

In order to investigate the behavior of the powder if the dwell time were extended, a 200 MPa pellet was produced with a total cycle duration of 60 s. In this case, the dwell time was 49 s, during which the punch separation was kept constant. The punches' displacement and upper punch force are reported in Figure S1 *a*. It is clear that the powder experienced a stress relaxation with a rapid drop of the stress in the first second, while the rest of the dwell time witnesses a slower decrease. The force transmitted to the upper punch after 1 second is 4.7 kN and corresponds to a stress of 166 MPa, while the load after 50 seconds decreases to 4.4 kN (155 MPa). These drops are important in view of the compaction force but will not be further discussed in this study.

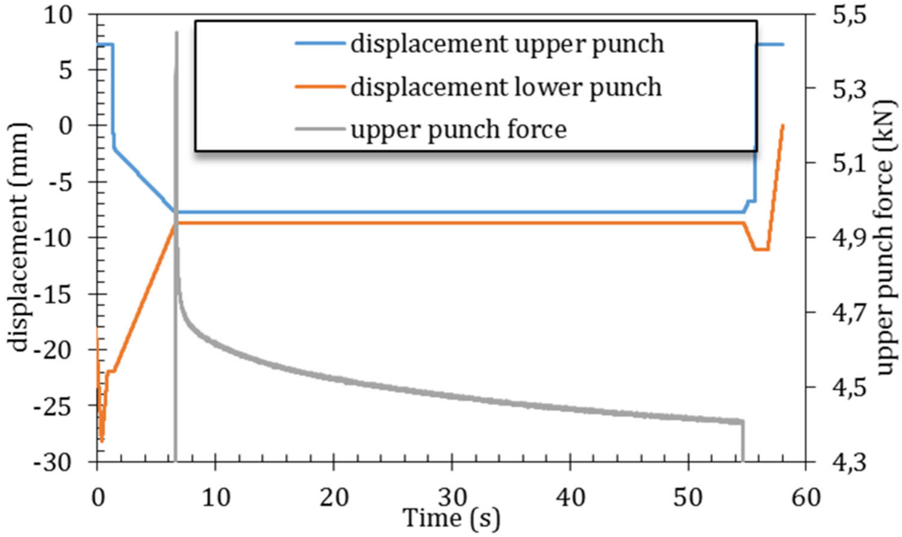

Figure S1: Compression test at 200 MPa with a 60 s cycle.

Thermal analyses and Raman spectra

With TGA analyses, DTA analyses were conducted. The results are shown in Figure S2 and present similarities between the powder and the pellets but also a major difference that will be discussed below. Analysis of the DTA curve of the powder has been described in-depth in our previous study. Briefly, the endothermic peak in the range [25 - 200 °C] corresponds to the loss of poorly bound water, and the underlying endothermic wide peak in the [200 – 350 °C] temperature range corresponds to the loss of structural water. The peak at 336 °C has been attributed to the condensation of HPO_4^{2-} to $P_2O_7^{4-}$ ions, and the narrow peak at 700 °C corresponds both to the crystallization of TCP and CPP phases. This analysis (consistent with the TGA) has been confirmed using XRD on the heated samples. Relative to the consolidated sample, the heat flow pattern looks the same except for the wide peak of the HPO_4^{2-}condensation at 336 °C. However, the Raman spectra of the ACP 25 MPa before and after TGA/DTA analyses up to 1000 °C presented in Figure S2 **Error! Reference source not found.**reveal the presence of an undeniable CPP phase with the signature bands at 1045 cm^{-1} and 736 cm^{-1}. The presence of β-TCP is confirmed with the bands at 950 and 970 cm^{-1}. As pyrophosphate ions' specific bands were not present in the spectrum of the 25 MPa consolidated sample before DTA, this implies that the formation of $P_2O_7^{4-}$ ions occurs during the heating of the sample. Consequently, this condensation might take place during a wider range of temperature, flattening the peak and making it difficult to observe. This increased range of temperature indicates that the condensation might be easier to trigger in the compact as it needs less thermal energy to start. This can be associated to a closer proximity of the HPO_4^{2-} ions in the pellet, making the condensation easier.

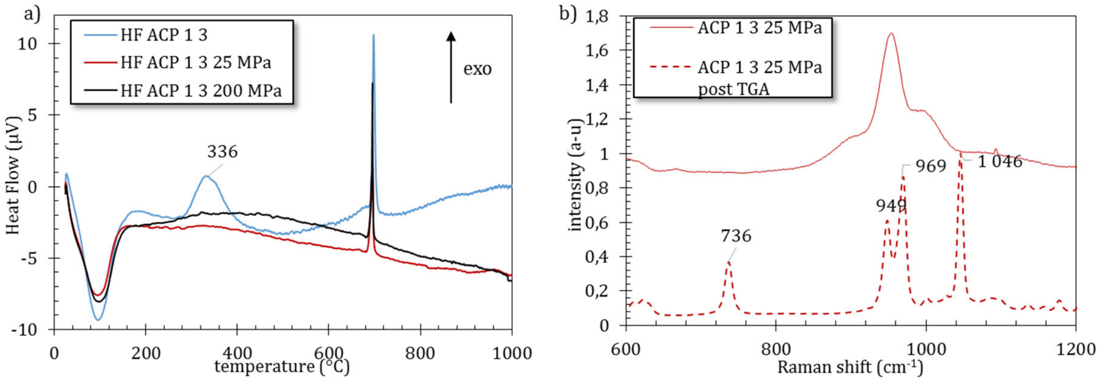

Figure S2 a) DTA analyses of the powder and the 25 MPa and 200 MPa pellets and b) Raman spectra of the 25 MPa pellet before and after TGA/DTA analysis.

SEM

Figure S3 presents the SEM images of the side of the pellet after the Brazilian test. Under low vacuum observation, the samples present a smooth surface and a profile characteristic of a Brazilian test break. However, when observed under higher vacuum (1.10^{-5} Pa), the samples presented a cracked surface and brittle mechanical properties. Unfortunately, no measurements were possible after vacuum exposition as the sample crumbled. This indicated the importance of the amount of water in the pellet concerning its mechanical properties. In fact, all samples were easily to handle except after a vacuum exposition.

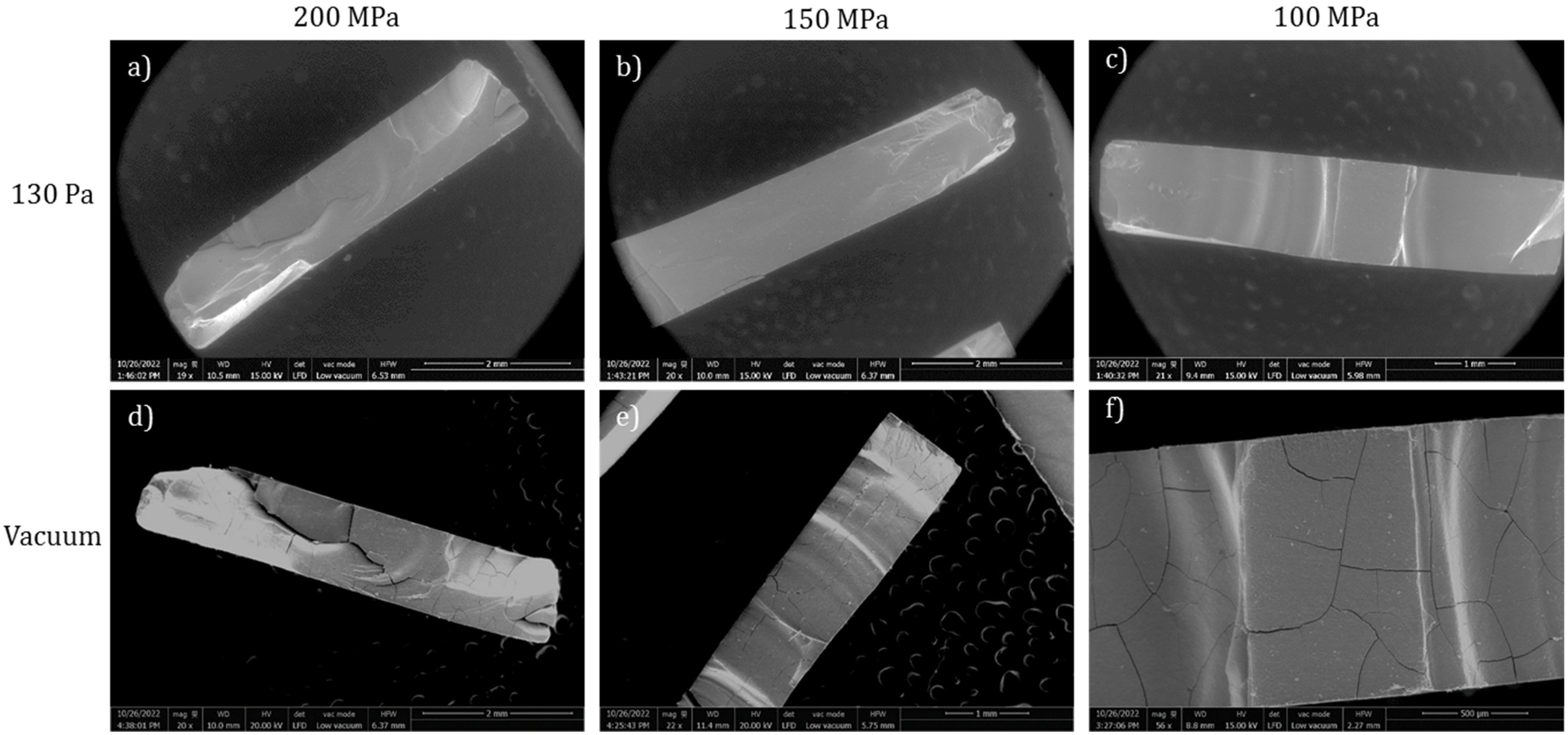

Figure S3: SEM pictures of 100, 150 and 200 MPa samples in 130 Pa and vacuum.
